# c-Jun promotes neuroblastoma cell differentiation by inhibiting APC formation via CDC16 and reduces neuroblastoma malignancy

**DOI:** 10.1186/s13062-025-00630-1

**Published:** 2025-03-27

**Authors:** Yunyun Wang, Jingjing Huang, Zhenhua Song, Shuo Zhang, Haojie Guo, Qi Leng, Na Fang, Shaoping Ji, Jian Yang

**Affiliations:** 1 Department of Biochemistry and Molecular Biology, Cell Signal Transduction Laboratory, School of Basic Medical Science, Henan University, Kaifeng, Henan Province 475004 China; 2 Zhengzhou Shuqing Medical College, Zhengzhou, Henan Province 450064 China; 3 College of Pharmacy and Nutrition, University of Saskatchewan, 107 Wiggins Road, Saskatoon, SK S7N 5E5 Canada

**Keywords:** c-Jun, CDC16, Anaphase-promoting complex, Cell differentiation, Neuroblastoma

## Abstract

Neuroblastoma is a pediatric embryonal malignancy characterized by impaired neuronal differentiation. Differentiation status in neuroblastoma strongly affects the clinical outcome, thus, enforcement of differentiation becomes a treatment strategy for this disease. However, the molecular mechanisms that control neuroblastoma differentiation are poorly understood. As an extensively studied protein of the activator protein-1 (AP-1) complex, c-Jun is involved in numerous cell regulations such as proliferation, survival and differentiation. In the current study, we demonstrated that c-Jun expression was upregulated by retinoic acid (RA) and flow cytometry assay indicated c-Jun overexpression arrested cell cycle to G1 phase, which, in turn, promoted the initiation of neuroblastoma cell differentiation. Co-immunoprecipitation (co-IP) assay showed that c-Jun competitively interacted with CDC16, a key subunit in anaphase-promoting complex (APC), resulting in reduced APC formation and inhibition of cell cycle progression. Furthermore, EdU proliferation assay and transwell experiment showed that c-Jun overexpression inhibited neuroblastoma cell proliferation and migration via interacting and sequestering CDC16. These findings identify c-Jun as a key regulator of neuroblastoma cell cycle and differentiation and may represent a promising therapeutic target to induce neuroblastoma differentiation via the interaction between c-Jun and CDC16.

**Supplementary Information:**

The online version contains supplementary material available at 10.1186/s13062-025-00630-1.

## Introduction

As early as a century ago, pathologists already discovered a link between cancer and the stagnation of cell differentiation. In general, poorly differentiated tumors tend to be more malignant, including childhood tumors especially neuroblastoma [1, 2]. Neuroblastoma is a childhood cancer of the developing sympathetic nervous system and the most common type of cancer in infancy. It develops from immature neuroblasts in the embryonic neural crest and is characterized by impaired neuronal differentiation [2, 3]. Histologically, neuroblastoma refers to a heterogeneous group of tumors, ranging from poorly differentiated neuroblasts to tumors containing more differentiated ganglion cells or ganglioneuroma-like components [1–5]. Neuroblastomas have been found to contain two cell types, namely adrenergic (ADRN) and mesenchymal (MES), which reflect different developmental stages of the sympathoadrenergic lineage, and where each cell type is maintained by a distinct core regulatory circuitry and set of super-enhancers [6]. The differentiation state has been recognized to be of prognostic significance for neuroblastoma. Patients with differentiating histology have significantly better survival than those with poorly differentiated neuroblastomas [4, 5]. Molecular basis for the positive correlation between neuroblastoma differentiation and favorable prognosis has been elucidated through recent studies, which highlight the roles of retinoic acid signaling, epigenetic regulation, and key transcription factors in promoting differentiation and improving outcomes [3–5]. Besides, positive correlation between neuroblastoma differentiation and favorable prognosis has been illustrated by microarray [7]. Thus, promoting cell differentiation has become a novel treatment strategy for this disease.

Retinoic acid (RA), which has been well studied, is an effective inducer of neuroblastoma cell differentiation [5, 8–10]. It can significantly improve event-free survival in patients with high-risk neuroblastoma [4, 5, 11, 12]. However, RA treatments are currently part of the treatment regimen for high-risk neuroblastoma, which has varied efficacy in patients and is limited to maintenance therapy for minimal residual disease, following aggressive chemo/radiotherapy [10, 11]. Resistance to RA has been observed in neuroblastoma patients and cell lines, rendering it a barrier to successful RA-based therapy [5, 9, 10, 12, 13]. A better understanding on the mechanism of neuroblastoma differentiation may provide novel ideas for differentiation-based therapy.

c-Jun is an important member of the basic transcription factor AP-1 family. It can form heterodimeric complexes with other Jun proteins such as JunB, JunD and c-Fos, and regulates the transcription of multiple genes that are involved in cell proliferation, survival, apoptosis and differentiation [14–18]. c-Jun is highly expressed in most tumors and promotes tumor cell survival, growth, migration and invasion [14, 19–21]. However, its expression is low in neuroblastoma cells. Overexpression of c-Jun enhanced the efficacy and persistence of CAR T cells against low-antigen tumor cells, implicating synergistic inhibitory effect of c-Jun in immunotherapy of neuroblastoma [22, 23].

c-Jun can play opposite roles in cell differentiation. Based on cell type, it may promote cell differentiation and inhibit cancer growth or inhibit cell differentiation and promote tumorigenesis [16, 24–27]. The role of c-Jun depends on the genetic background of cell types and the differentiation state of cells. Neuroblastoma, a prevalent pediatric neurological tumor, arises from neural crest-derived cells (NCCs) that undergo defective differentiation during development [4]. During neurodevelopment, c-Jun is involved in differentiation of neural stem cells, which is crucial for neuronal polarity [28–30]. The expression of c-Jun is low or even absent in normal nerves but rises rapidly after nerve injury, initiating a myelin repair program and promoting neuron regeneration and axon elongation [17, 18, 31–33]. DMSO (dimethyl sulfoxide), NGF (nerve growth factor) or RA-induced differentiation of neuroblastoma cells is associated with increased c-Jun expression [9, 16, 24, 27]. However, the roles of c-Jun in neuroblastoma cell differentiation and the underlying molecular mechanisms are not well understood. In the present study, we demonstrated that c-Jun is a regulator of neuroblastoma cell cycle and differentiation and can be a prognostic marker for survival in neuroblastoma patients.

## Materials and methods

### Cell line and culturing conditions

Human neuroblastoma cell line SH-SY5Y and human embryonic kidney cell line 293T were purchased from the American Type Culture Collection (ATCC, Manassas, VA, USA). Cell line SH-SY5Y was cultured in Dulbecco’s modified Eagle’s medium (MEM/F’12) (Thermo Scientific, HyClone, Logan, UT, USA) supplemented with 10% fetal bovine serum (FBS) (Thermo Scientific, Gibco, Grand Island, NY, USA) and 100 IU/mL penicillin and 100 µg/mL streptomycin (Solarbio, Beijing, China). Cell line 293T was cultured in Dulbecco’s modified Eagle’s medium (DMEM) (HyClone) supplemented with 10% FBS (Biological Industries USA, Cromwell, CT, USA) and 100 IU/mL penicillin and 100 µg/mL streptomycin (Solarbio). Cells were maintained in a humidified atmosphere containing 5% CO_2_ at 37 ˚C.

### Construction of stable c-Jun overexpression cells

Lentiviral vector plenti-c-Jun-flag-puromycin was designed and constructed by GeneChem (Shanghai, China) for stable c-Jun-overexpression (c-Jun OE) cell line and its negative control (NC, transfected with Lentiviral vector plenti-flag-puromycin). In addition, c-Jun short hairpin RNA (shRNA) was cloned into GL401‐lentiviral vector (pCLenti-U6 -c-Jun shRNA-CMV-Puro-WPRE) by Obio Biotechnology (Shanghai, China) for c-Jun‐knock down (c-Jun KD) cell line. GL401NC (pCLenti-U6-shRNA(NC)-CMV-Puro-WPRE) were used as negative control. Target sequences of shRNA were listed in supplemental Table S1. Then, recombinant c-Jun lentiviruses were transfected into SH-SY5Y cells according to manufacturer’s instructions. 2 µg/mL puromycin (Ameresco, Framingham, MA, USA) was employed to filter stable transfection cells for three days. Western blot and quantitative reverse‐transcription PCR (qRT-PCR) were applied to validate efficacy of transfection. Eventually, stable transfection cell with best efficacy was picked for further experiments.

### Construction of CDC16 overexpression and CDC16 ShRNA plasmid

Lentiviral vector plenti-CDC16-flag-puromycin was designed and constructed by GeneChem (Shanghai, China) for CDC16 overexpression and the CDC16 overexpression and control lentiviruses were provided by GeneChem (Shanghai, China). Then, c-Jun OE cells were infected with CDC16 and control lentiviruses for further detection.

The siRNA sequences for CDC16 were designed using DSIR (http://biodev.extra.cea.fr/DSIR/DSIR.html). The sequence with the highest score was selected. After incorporating elements such as loop sequences and restriction sites, it was cloned into the pLVX-shRNA2-Puro vector for CDC16-knock down (CDC16 shRNA). c-Jun OE cells were transfected with CDC16 siRNA and control siRNA using the Lipo8000 transfection for further detection. Target sequences of siRNA and shRNA were listed in Supplementary Table 1.

### RNA preparation, cDNA synthesis and real-time PCR

Total RNA was isolated from SH-SY5Y cells by Trizol (Takara Bio Inc., Beijing, China), and then reverse transcribed into cDNA using reverse transcriptase from Vazyme (Nanjing, Jiangsu, China) and random primers (Shenggong Bioengineering Co., Zhengzhou, Henan, China). SYBR Green (Takara Bio Inc., Shiga, Japan)-based real-time PCR was performed using an ABI 7900 thermocycler (Thermo Fisher Scientific, Waltham, MA, USA). The reactions were incubated in a 96-well plate at 95 °C for 10 min followed by 40 cycles of 95 °C for 15 s and 60 °C for 30 s. Quantitative PCR primers were shown in Supplementary Table 2. The Ct value was measured during the exponential amplification phase. The relative expression levels of target genes were given by 2^−ΔΔCt^ and log_2_FC values were presented as the relative changes compared to the controls.

### Western blot

For each Western blot assay, cultured cells were collected, washed with PBS, and lysed in radioimmunoprecipitation (RIPA) assay buffer. The cell lysate was then centrifuged (12,000 rpm) at 4℃ for 5 min. Concentration of the extracted protein were quantified using BCA™ Protein Assay Kit (CoWin Biosciences, Taizhou, Jiangsu, China). Samples containing equal amount of proteins were separated by 10% Bis-Tris gels using a Bio-Rad Bis-Tris Gel System (Bio-Rad Laboratories, Hercules, CA, USA) and then transferred onto a polyvinylidene difluoride membranes (PVDF) membrane (Millipore, MA, USA). The membrane was blotted with 5% nonfat milk and incubated with primary antibodies (1:1000) overnight at 4℃. The membrane was then washed with TBST 3 times (10 min each time) to remove non-specific antibody bindings. After washing, the membrane was incubated with horseradish peroxidase (HRP)-labeled secondary antibodies (1:5,000) (Beyotime Biotechnology, Shanghai, China) at room temperature for 1 h and subsequently washed with TBST. Finally, the antigen-antibody conjugates were detected by the enhanced chemiluminescence (Meilunbio Co., Dalian, Liaoning, China). The current Western blot assay included antibodies against c-Jun (Thermo Scientific-Pierce, Rockford, IL, USA); CDC16, MMP-2, MMP-9, TIMP-2, cyclin D1,cyclin A2, cyclin E1, CDK2,CDK4, β-catenin, N-cadherin, E-cadherin, vimentin, p21, CDC20 and GAPDH (Proteintech, Wuhan, Hubei, China); and GSK3β and p-GSK3β(S9) (Cell Signaling Technology, Shanghai, China).

### SiRNA transfection

Cells growing in the logarithmic phase were seeded in 6-well plates (6–8 × 10^5^ cells/mL) and cultured for 24 h for attachment. Upon reaching 60–70% confluency, the cells were transfected with siRNAs using Lipofectamine 3000 reagent (Invitrogen, Shanghai, China) following protocol recommended by the manufacturer. The plates were then incubated at 37 °C for 6 h before culture media being changed to MEM/F’12 containing 10% FBS for subsequent experiments. Efficacy of the transfection was verified using Western blot.

### Scratch test

Cells growing in the logarithmic phase were seeded in a 6-well plate and allowed to grow to 70–80% confluency. Then, cell culture medium was discarded, and the cell layer was scratched vertically and horizontally using 10 µL pipette tips. After washing with PBS twice, initial scratches were recorded using an inverted phase contrast microscope. Images were also acquired 12 h and 24 h after scratching. Pictures were acquired from 3 to 5 areas of each well, and at least three independent experiments were performed. Cell migration distance was measured and calculated using ImageJ.

### Cell migration and invasion assay

Invasion and migration were assayed in triplicate experiments using 24-well plates with Transwell inserts (Corning Incorporated, New York, NY, USA). The Transwell inserts were outfitted with sterilized polyethylene terephthalate filtration membrane containing 8-micron pores. The membrane was coated with 100 µL Matrigel (BD Biosciences, San Jose, CA, USA) and serum-free MEM/F-12 at a 1:4 dilution for 8 h at 37℃. A total of 2 × 10^4^ cells in serum-free medium was added to the upper chamber of the Transwell insert, and 0.8 mL of MEM/F-12 containing 20% FBS was added to the lower chamber. Cells were incubated in the Transwell inserts at 37 ◦C and 5% CO_2_ for 5 h. Then, the migration and invasion assays were performed after the corresponding period. Finally, invaded and migrated cells were fixed with 4% paraformaldehyde for 20 min and stained with 1.5% crystal violet for 30 min. The invaded or migrated cells were observed under a microscope with images captured.

### Cell proliferation assay

Cell proliferation was determined using the EdU incorporation assay kit (Ribobio Co., Guangzhou, Guangdong, China) following protocol recommended by the manufacturer. Briefly, SH-SY5Y cells were seeded into a 96-well plate (5 × 10^4^ cells/per well) and cultured, and EdU (50 mM) was added for 12 h before the end of the culture to label proliferating cells. Then, the cells were fixed by 4% paraformaldehyde (Sigma-Aldrich, Wuxi, Jiangsu, China) for 30 min. Apollo reaction cocktail was added for 30 min, and Hoechst 33,342 was used for DNA staining. Analysis of SH-SY5Y cells proliferation, represented as the ratio of EdU^+^ to total cells, was performed using representative fluorescence images.

### Evaluation of cell viability

Cell counting kit-8 assay (CCK-8; Beyotime Biotechnology, Shanghai, China) was used to evaluate viability of the SH-SY5Y cells. Briefly, for each individual exposure time, SH-SY5Y cells were seeded into a 96-well plate (1 × 10^4^ cells/well) and cultured. CCK-8 solution (10 µL) was added into each well of the plate 2 h prior to the end point of treatment. The plate was then incubated at 37 °C for 2 h before the absorbance at 450 nm was recorded for each well using a microplate reader (Bio-Tek, Winooski, VT, USA).

### Induction of cell differentiation

SH-SY5Y cells was seeded into a poly-D-lysine coated 6-well plate (2 × 10^5^ cells/well) and subsequently cultured for 9 d or 15 d. During culture, differentiation induction reagents such as RA and BDNF were added. Briefly, the differentiation medium supplemented with 10 µM RA (Merck, MA, USA) was used to induce cell differentiation, with media changes every two days. After five days of induction, the medium was replaced with differentiation medium containing 50 ng/mL BDNF(SinoBiological, Beijing, China), and the medium were changed every two days until the 15th day of induction. Differentiation of SH-SY5Y cells was evaluated by the level of differentiation-related protein markers including GAP43 and β-III-tubulin, immunofluorescent staining and Western blot after 9 d or 15 d of treatment.

### Immunofluorescent staining

Cells were uniformly seeded onto coverslips at 1 × 10^5^ cells/mL with differentiation being induced by RA treatment for 3 d or 9 d before being fixed with 4% paraformaldehyde. After fixation, 100 µL Triton X-100 (0.5%) was added and the coverslips were incubated at room temperature for 20 min. Then, the coverslips were washed twice with PBS, blocked with 3% BSA for 30 min, and incubated with TUBB3 antibody (1:200) at 4℃ overnight. Next day, the coverslips were incubated with Alexa Fluor-555 anti-mouse secondary antibody at room temperature for 50 min. Nuclei were counter-stained with DAPI for 10 min, and the coverslips were mounted with an anti-fluorescence quenching mounting solution.

Fluorescence images were acquired using a Nikon fluorescence microscope with appropriate excitation wavelengths. Images were captured at 10× magnification, adjusting exposure time and gain to avoid overexposure or signal saturation. At least 5 regions for each coverslip were imaged to ensure representative data.

Images were processed in ImageJ with background subtraction and contrast enhancement to improve signal visibility. Cell counts were performed using cell counter plugins. Thresholding was applied to exclude background signals, and cells with neurite were identified, the percentage of cells with neurite were calculated for each image, and then the average percentage of cell with neurite on each coverslip were analyzed (at least 3 coverslips for each group, *n* ≥ 3). Statistical analysis was performed using GraphPad Prism8.0. Data are presented as mean ± standard deviation, with statistical significance determined by t-tests or ANOVA (*p* < 0.05).

### Flow cytometry analysis

Cell cycle distribution was analyzed by flow cytometry after cell transfection and 48 h incubation. Briefly, cells were collected according to the protocol for the Cell Cycle Analysis Kit (Shanghai Enzyme-linked Biotechnology Co. Ltd, Shanghai, China). After incubation, the cells were analyzed by a Beckman CytoFLEX flow cytometer (BeckmanCoulter, Shanghai, China). For cell cycle analysis, cells were stained with Propidium Iodide (PI), and the FL2 (PI) channel was selected to measure DNA content. The analysis model was chosen based on the data characteristics, such as single-peak distribution. The Cell Cycle analysis tool in CytoExpert was used to perform DNA content distribution analysis. Data were imported into CytoExpert software, and a quality check was performed to exclude debris, doublets, dead cells, and abnormal data points. Appropriate gates were set using the FSC/SSC scatter plot to select the live cell population. The resulting DNA content distribution plot showed the G0/G1, S, and G2/M phases, and the percentage of cells in each phase was recorded.

### Co-immunoprecipitation assay (Co-IP)

Co-IPs were performed with Dynabeads Protein G (Thermo Fisher Scientific, Shanghai, China) following the protocol recommended by the manufacturer. Briefly, 1.5 mg Dynabeads was conjugated with 5 µg IgG or anti-CDC16 antibody (Proteintech, Wuhan, Hubei, China) at 4 °C overnight. Next day, total cell lysates and the antibody-conjugated Dynabeads were mixed and incubated at 4℃ overnight with shaking. After washing three times with PBS containing 0.1% Tween-20, the beads were boiled at 100 °C for 10 min with the 2×Protein loading buffer and the supernatant was collected for WB analysis.

### Statistical analysis

All experiments in this study were carried out at least in triplicate. GraphPad 8.0 software (GraphPad, San Diego, CA, USA) was used for statistical analysis and the results were presented as the mean ± SEM. The *p* values were calculated using one-way analysis of variance (ANOVA) or Student’s *t*-test. A value of *p* < 0.05 was statistically significant.

## Results

High c-Jun expression is associated with neuroblastoma differentiation.

**Fig. 1:**
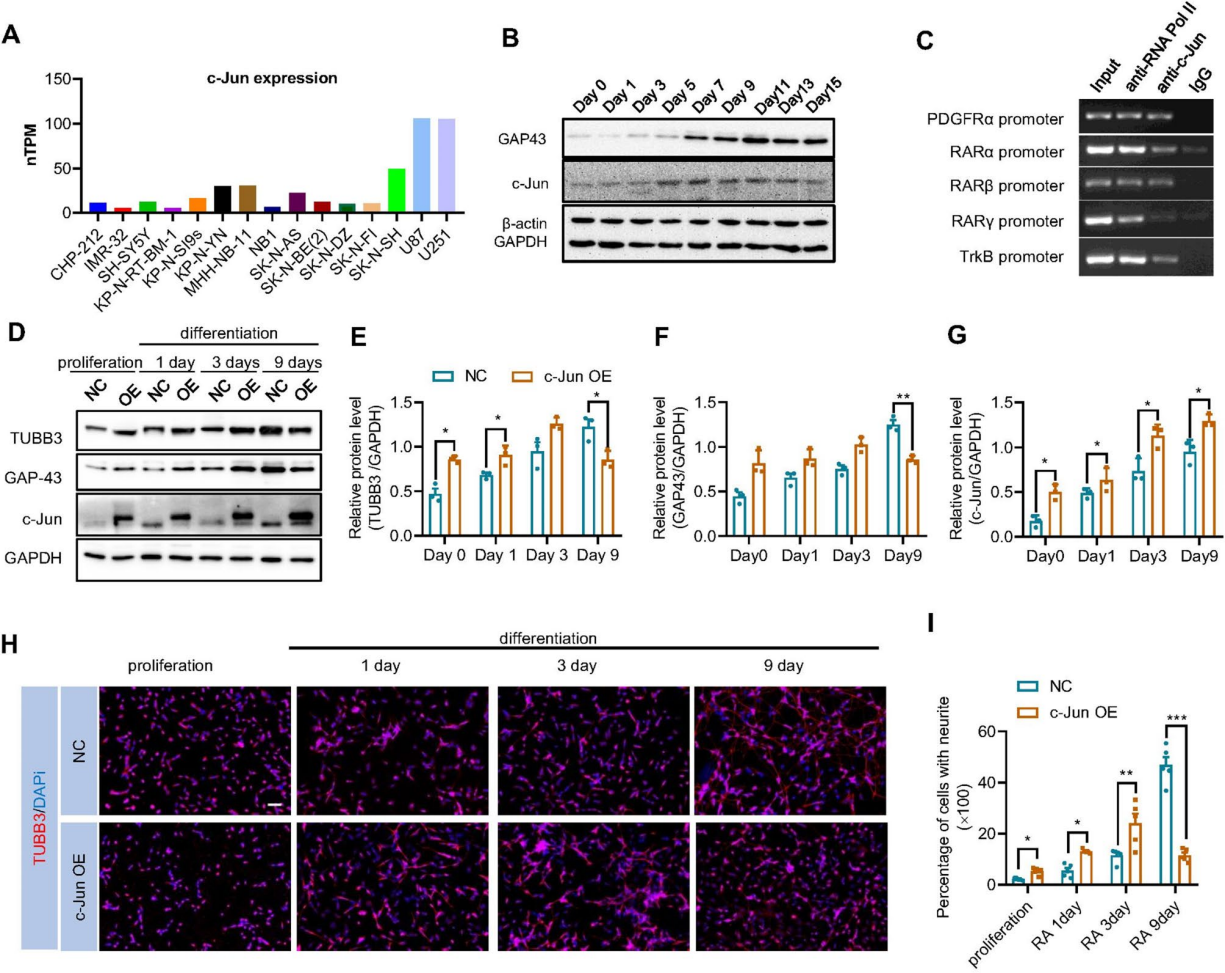
c-Jun induced neuroblastoma cell differentiation. **(A).** c-Jun expression in different neuroblastoma cell lines and glioblastoma cell lines deposited at The Human Protein Atlas. **(B)**. SH-SY5Y cells were induced for differentiation by RA and expression of c-Jun protein during differentiation was detected by Western blot (*n* = 3). **(C).** Ch-IP assay by using c-Jun antibody to detect the binding of c-Jun onto the promoters of PDGFRα, RARα, RARβ and RARγ. **(D).** Immunoblots showing the expression of differentiation markers in control cells and c-Jun OE cells after RA-induced differentiation. **(E-G).** Relative protein level of TUBB3, GAP43 and c-Jun were analyzed by unpaired *t*-test (mean ± SEM of triplicate experiments). *p < 0.05, **p < 0.01. **(H).** Neural differentiation of negative control (NC, cells transfected with Lentiviral vector) and c-Jun OE SH-SY5Y cells. Cells were stained with TUBB3 and DAPI, and images were acquired using a fluorescence microscope (Nikon). **(I).** Number of cells with neurite outgrowth (> 20 μm) was counted using Image J software. Pictures were taken from 3–5 areas of each well with at least three independent experiments. The mean of each well was used for statistical analysis with unpaired *t*-test. Means with SEM were shown. *p < 0.05, **p < 0.01, ***p < 0.001

Differentiation of human neuroblastoma cells including SH-SY5Y cells can be induced by retinoic acid (RA), a known neuronal differentiation inducer used as a clinical therapeutic [1, 34]. According to data deposited at The Human Protein Atlas (HPA), expression of c-Jun is low or even absent in most neuroblastoma cells, especially SH-SY5Y (Fig. 1A). However, its level rose upon RA-induced differentiation and kept on increasing along the progression of differentiation (Fig. 1B). In addition, the Ch-IP assay showed that c-Jun bound to RARβ, TrkB and PDGFRα promoters, which are crucial to RA-induced neuroblastoma cell differentiation [8, 35–38] (Fig. 1C). These observations suggest that c-Jun is likely to play an important role in neuroblastoma cell differentiation.

To study the functional role of c-Jun in neuroblastoma cell differentiation, we constructed a stable cell line with high c-Jun expression since its expression is low in undifferentiated SH-SY5Y cells. c-Jun overexpression was confirmed by Western blot and qPCR (Supplementary Fig. 1A-B). After differentiation induced by RA and brain-derived growth factor, both mRNA and protein expressions of differentiation-related markers GAP43 and TUBB3 were significantly increased compared to the control (NC) at the initial differentiation stage (0-3 day) in c-Jun overexpressed(c-Jun OE) SH-SY5Y cells (Fig. 1D-G, protein; and Supplementary Fig. 1C-E, mRNA). Furthermore, c-Jun overexpression led to prominent SH-SY5Y cell spreading and neurite outgrowth (Fig. 1H-I). Interestingly, the expression of the neuronal markers were reduced at day 9 of differentiation (Fig. 1D-G and Supplementary Fig. 1F), suggesting a different regulatory role of c-Jun along the progression of differentiation. From the ChIP assay, we observed that c-Jun bound to its own promoter (Supplementary Fig. 1G), implying a self-regulation mechanism.

### C-Jun induces G1 arrest and represses cell cycle-promoting genes

**Fig. 2:**
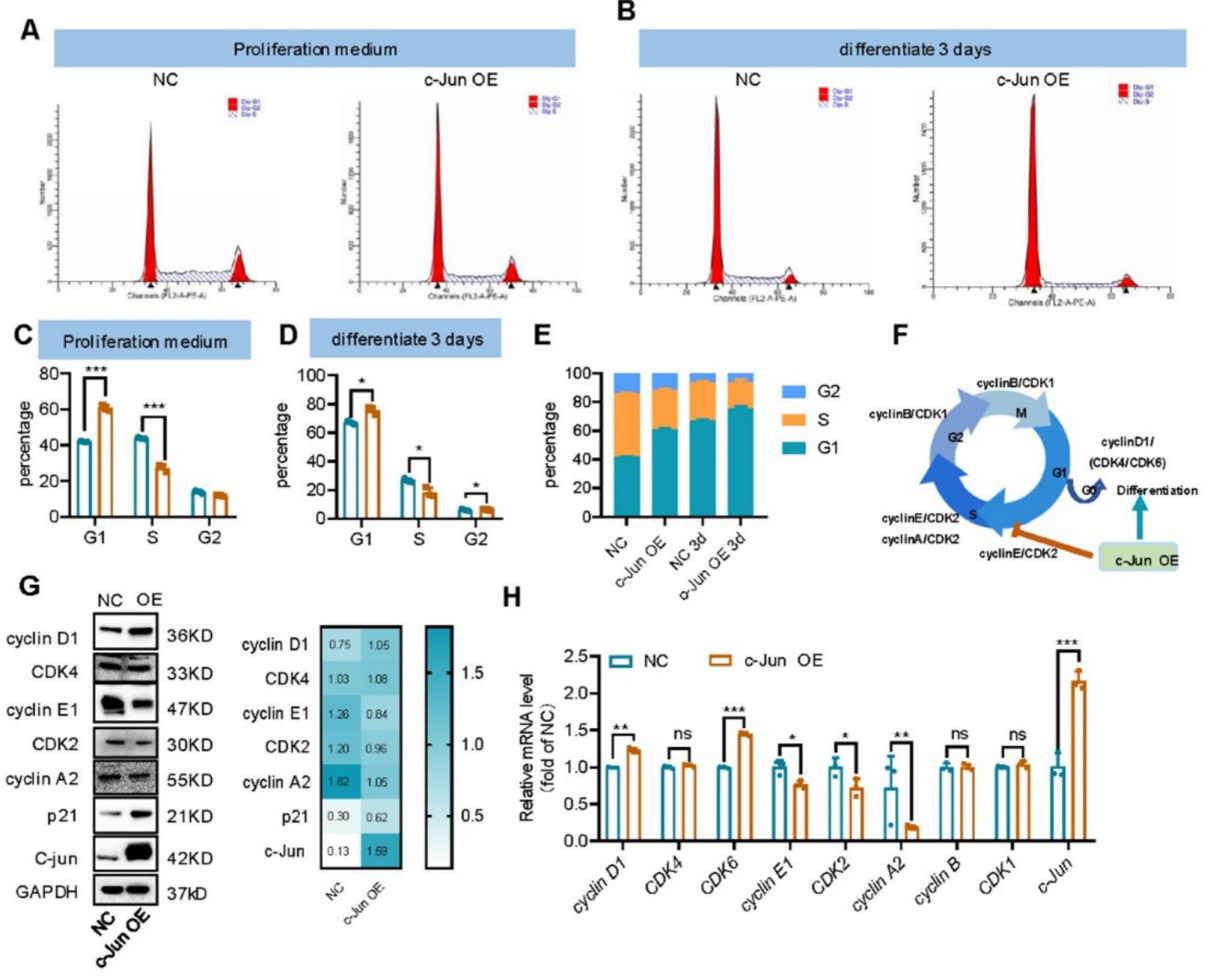
c-Jun overexpression resulted in cell cycle arrest in G1 stage. **(A-E).** Cell cycle of neuroblastoma cell lines analyzed by flow cytometry (*p < 0.05, ***p < 0.001, and bar graphs represent the mean ± SEM). **(F).** Scheme of cell cycle progression and cyclins expression at different stages. c-Jun overexpression inhibited cell cycle progression from G1 to S stage and promoted G1 to G0 and subsequent cell differentiation. **(G).** Expression of cell cycle related markers in negative control (NC) cells and c-Jun overexpressed (c-Jun OE) cells measured by Western blot. Relative protein level of were analyzed and heatmap showed mean ± SEM of triplicate experiments. **(H).** qPCR determination of the mRNA expression of cell cycle-related markers (*cyclin D1*,* cyclin E1*,* cyclin A2*,* cyclin B and CDKs*) in negative control (NC) cells and c-Jun overexpressed (c-Jun OE) cells. Data were analyzed with unpaired t-test (mean ± SEM of triplicate experiments). *p < 0.05, **p < 0.01, ***p < 0.001

The above study showed that c-Jun plays a vital role in the initiation of SH-SY5Y cell differentiation. Since onset of differentiation is assumed to follow the withdrawal from the cell cycle [39, 40] and neuroblastoma is a disease of abnormal cell differentiation, we performed flow cytometry to evaluate cell cycle changes in SH-SY5Y cells infected with negative control (NC) or c-Jun overexpressed (c-Jun OE) lentivirus. As shown in Fig. 2A, C and E, 60.7% of the c-Jun OE cells were arrested in the G1 phase,18% more than NC cells (*p* < 0.0001). Only 27.3% of the c-Jun OE cells were in S phase, 17% less than the NC cells (*p* < 0.001). No significant difference was observed between the two types of cells in G2 phase. Furthermore, we measured cell cycle changes under differentiation condition of 0.3 µg/mL RA treatment (Fig. 2B, D and E). For the NC cells, 67.0% were arrested in the G1 phase, 23% higher than the proliferation condition, and 26.7% were in S phase, 17.3% less than the proliferation condition. As for the c-Jun OE cells, 8.5% more were arrested in the G1 phase and 8.4% less were in S phase, respectively, compared to the NC cells. This implicates that c-Jun overexpression increases G1 arrest and reduces G1/S transition, resulting in downregulation of S phase and decreased DNA synthesis (Fig. 2A). Since c-Jun is a transcription factor that can regulate the expression of cell cycle related proteins [41–43], we further analyzed gene expression profiles of the SH-SY5Y cells with or without c-Jun overexpression. In c-Jun OE cells, cyclin D1, a marker for G1 phase [44], was significantly upregulated. However, cyclin E1, cyclin A2 and cyclin B, required for the cell cycle progression from S to M [45], were downregulated (Fig. 2G-H). In addition, p21 protein, which represses cell cycle, was upregulated (Fig. 2G). Transcription of cyclin genes is regulated in a cell cycle dependent manner; thus, downregulation of the cyclins could be a consequence of G1 arrest. In conclusion, c-Jun overexpression can induce G1 arrest and promote initiation of cell differentiation.

### Overexpression of c-Jun inhibits proliferation and migration of neuroblastoma cells

**Fig. 3:**
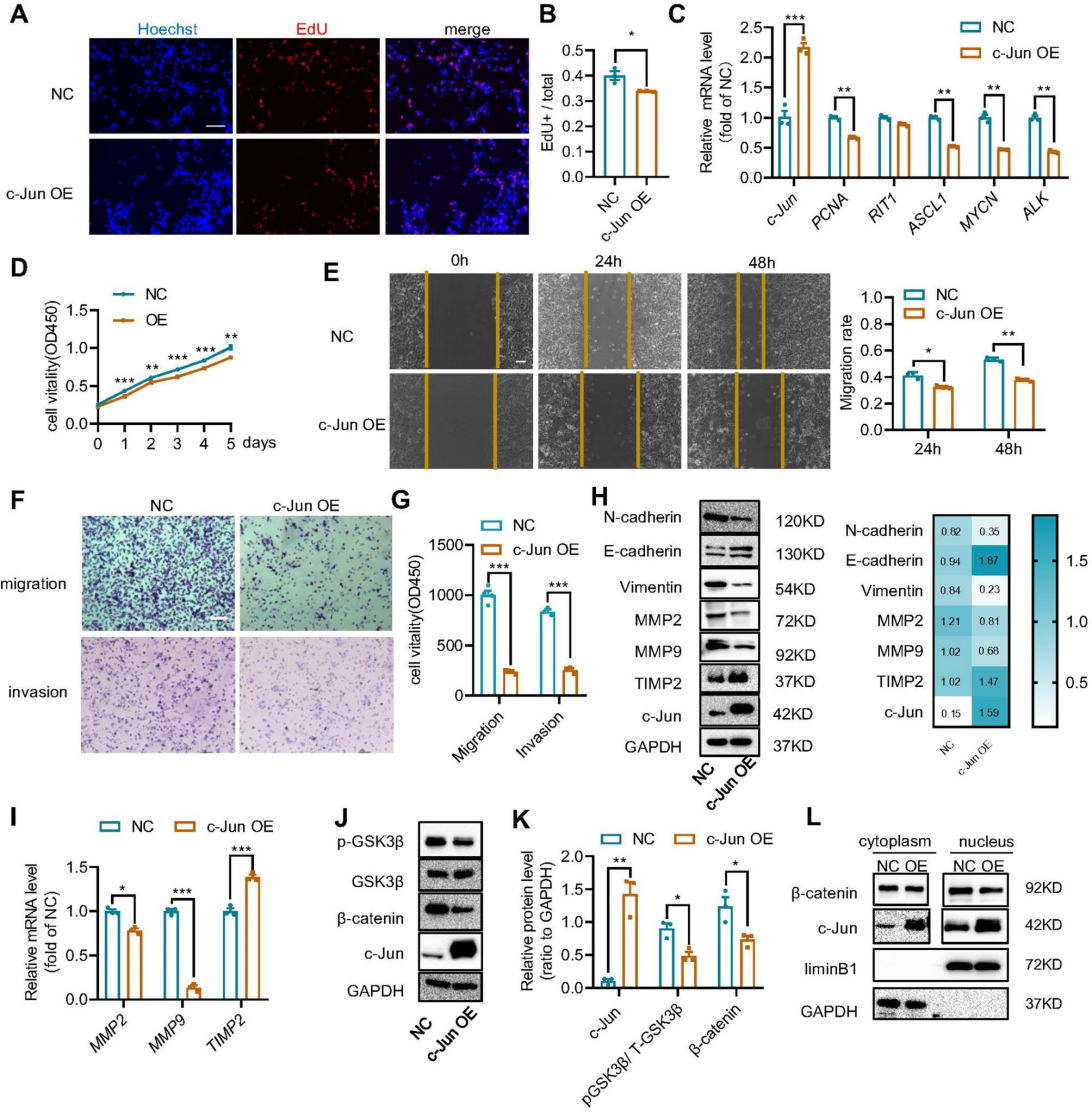
c-Jun overexpression inhibited SH-SY5Y cell proliferation and migration. **(A).** EdU proliferation assay of negative control (NC) and c-Jun OE SH-SY5Y cells with representative images shown. **(B).** Calculated ratio of number of EdU + cells to total number of cells. Data were represented as mean ± SEM of three independent experiments with mean number collected from five areas of each well. **(C).** qPCR assay to detect the mRNA level of proliferation related genes in NC and c-Jun OE SH-SY5Y cells. **(D).** Viability of NC and c-Jun OE cells measured with CCK-8 assay. **(E).** Cell scratch experiment to evaluate the effect of c-Jun overexpression on wound healing ability of SH-SY5Y cells. Migration rate was statistically analyzed with the ratio of migrated area to original scratched area. **(F-G).** Transwell assay to evaluate the effect of c-Jun overexpression on cell migration and invasion. Migrated cell number were calculated and statistically analyzed by unpaired *t*-test. **(H).** Protein immunoblotting assay to detect changes of EMT and migration-related proteins (N-cadherin, E-cadherin, vimentin, MMP9, MMP2 and TIMP2). Results were presented as mean ± SEM (*n* = 3). **(I).** qPCR to examine mRNA changes of migration-related genes (*MMP9*,* MMP2 and TIMP2*) in c-Jun OE cells. qPCR results were calculated using 2^−ΔΔCT^ and analyzed by Graphpad software. The results were presented as mean ± SEM (*n* = 3). **(J).** Immunoblot assay to detect phosphorylation level of GSK3β at ser9 and expression level of β-catenin. **(K).** Relative protein levels of c-Jun, pGSK3β and β-catenin were determined by the ratio to GAPDH. **(L).** Changes of β-catenin in nucleus as detected by nucleocytoplasmic separation experiment. All data were statistically analyzed with unpaired *t-*test, *p < 0.05, **p < 0.01, ***p < 0.001. OE represents SH-SY5Y cells infected with c-Jun overexpression lentivirus, whereas NC represents SH-SY5Y cells infected with control virus. Scale bar = 100 μm

For most tumor cells, G1 arrest and initiation of cell differentiation are usually accompanied by a decrease of cell proliferation [46, 47]. Thus, we evaluated the effect of c-Jun overexpression on the proliferation of SH-SY5Y cells using EdU proliferation and CCK-8 assays. Compared with the control group, the proportion of EdU + cells was significantly reduced in the c-Jun OE SH-SY5Y cells (Fig. 3A-B) and increased in the c-Jun knockdown SH-SY5Y cells (Supplementary Fig. 2A-B). This implicates that c-Jun overexpression can suppress the proliferation of SH-SY5Y cells. We further measured mRNA changes of proliferation-related genes in c-Jun OE SH-SY5Y cells by qPCR. Transcription factors *PCNA*,* ASCL1*,* RITI*,* MYCN* and *ALK* were downregulated, which reinforces that c-Jun inhibits the proliferation of SH-SY5Y cells (Fig. 3C). The CCK-8 assay also confirmed that c-Jun overexpression decreased SH-SY5Y cell viability (Fig. 3D).

Since c-Jun promotes neuroblastoma differentiation, which is associated with reduced malignancy, we investigated the impact of c-Jun overexpression on tumor cell migration, a key hallmark of malignancy. First, migration ability of the SH-SY5Y cells was evaluated using both Wound-healing and Transwell assays. c-Jun overexpression inhibited migration and invasion of the SH-SY5Y cells (Fig. 3E-G), whereas c-Jun knockdown enhanced migration and invasion of the SH-SY5Y cells (Supplementary Fig. 2C-E). Furthermore, mRNA and protein expression of migration markers, such as MMP2, MMP9 and TIMP2, were analyzed to assess changes in migratory behavior [48, 49]. In c-Jun OE SH-SY5Y cells, MMP2 and MMP9 were down-regulated whereas TIMP2 was up-regulated (Fig. 3H-I and Supplementary Fig. 2F), further supporting that high expression of c-Jun could inhibit the migration of SH-SY5Y cells.

Epithelial mesenchymal transformation (EMT) is the process by which epithelial cells transform into mesenchymal cells with the ability to migrate and invade. It is a key link in the migration and invasion of most cancers [50–52]. Expression levels of both epithelial and mesenchymal markers were evaluated in the SH-SY5Y cells. c-Jun overexpression upregulated E-cadherin but downregulated N-cadherin and vimentin (Fig. 3H-I), implying that c-Jun could inhibit the migration and invasion of SH-SY5Y cells via regulating EMT. The role of WNT signaling, a well-established regulator of cell migration and invasion in neuroblastoma [4, 53], was also examined. The results showed that c-Jun overexpression inhibited the expression of β-catenin inside the nuclei and decreased the phosphorylation level of GSK3β at Ser9(pGSK3β(s9)) (Fig. 3J-L), both of which play important roles in neuroblastoma cell proliferation and differentiation [53, 54].

### High c-Jun expression is prognostic for better survival in neuroblastoma patients

**Fig. 4:**
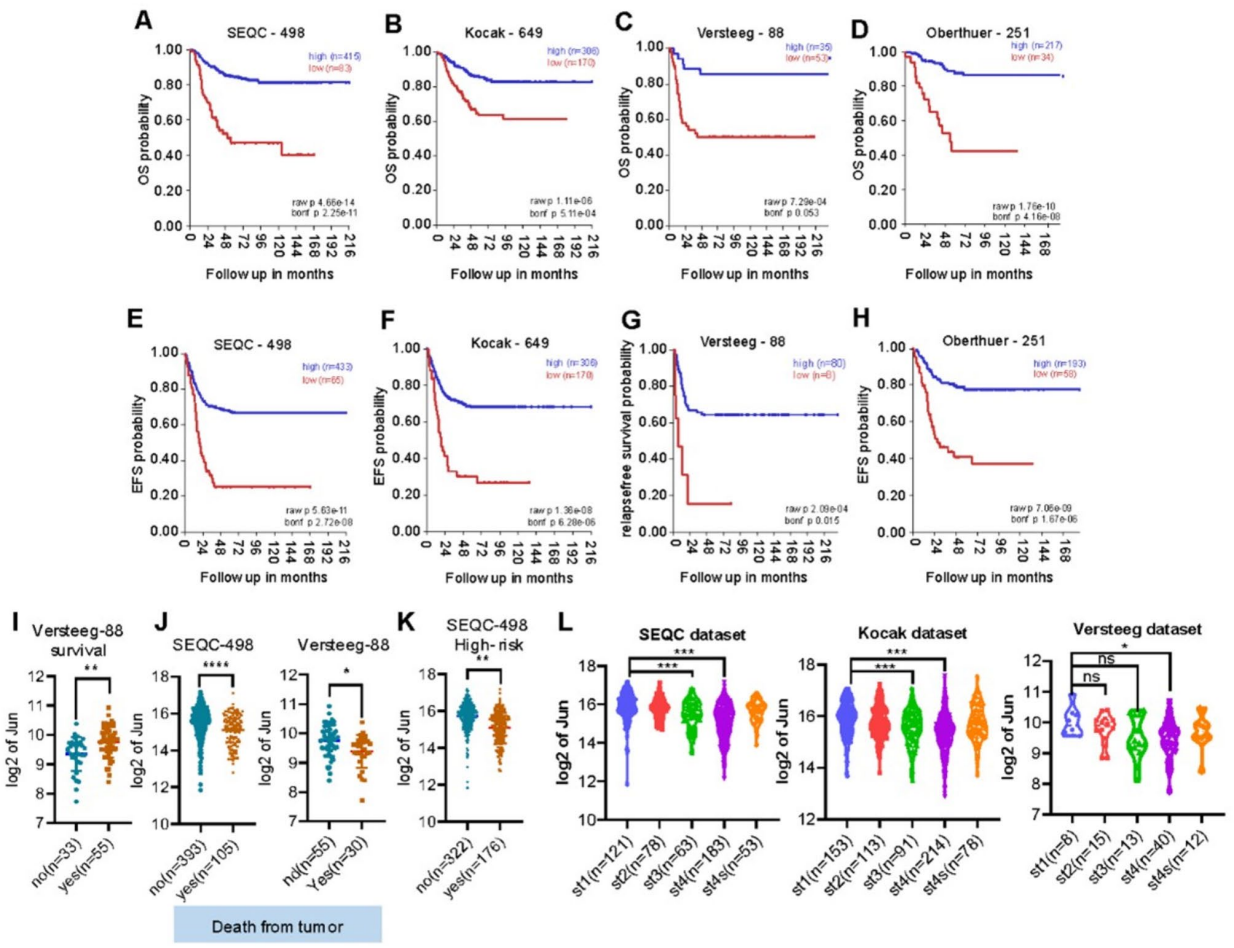
Prognostic significance of c-Jun in neuroblastoma. **(A-H).** Kaplan-Meier plots of overall survival (OS) and event-free survival (EFS) with *p* values corrected for multiple testing (Bonferroni correction) in four independent neuroblastoma patient cohorts, RPM/SEQC (*n* = 498; GEO: GSE62564), Kocak (*n* = 649; GEO: GSE45547), AMC/Versteeg (*n* = 88; GEO: GSE16476 88/122) and Oberthuer (*n* = 251; ArrayExpress: E-TABM-38). **(I).** Correlation of c-Jun expression with patients’ survival (*n* = 55) or not (*n* = 33) in AMC/Versteeg (*n* = 88; GEO: GSE16476 88/122). **(J).** Expression of c-Jun in patient death from tumor(yes) or not (no death, nd) in two different patient cohorts RPM/SEQC and AMC/Versteeg. **(K).** Correlation of c-Jun expression with high-risk of neuroblastoma (*n* = 176) or not (*n* = 322) in RPM/SEQC. Data in I, J and K were analyzed with unpaired t-test, *p < 0.05, **p < 0.01, ****p < 0.0001. **(L).** Box plot of c-Jun expression levels in stage (st) 1–4 S tumors in three database, RPM/SEQC, Kocak and AMC/Versteeg. Data were analyzed using one-way ANOVA with Dunnett’s multiple comparisons test, ***p < 0.001, ****p < 0.0001

Since high level of c-Jun is associated with neuroblastoma differentiation, we evaluated whether c-Jun could be a prognostic marker for neuroblastoma. Four neuroblastoma datasets (SEQC: GSE62564; Kocak: GSE45547; Versteeg: GSE16476 and Oberthuer: E-TABM-38) were analyzed. First, overall survival (OS) and event-free survival (EFS) were assessed by Kaplan Meier analysis. Patients with high c-Jun expression was associated with better OS and EFS (Fig. 4A-H). c-Jun expression is positively correlated with patient’s survival (Fig. 4I), and decreased c-Jun expression correlates significantly with death from disease (Fig. 4J) and high-risk (Fig. 4K). Furthermore, the relationship between c-Jun expression and clinical characteristics was analyzed and showed that the expression of c-Jun was negatively associated with the clinical stage of neuroblastoma. From stage I to stage IV, c-Jun expression decreases along with the clinical stage but escalated at stage 4s (Fig. 4L). Stage 4s neuroblastoma expressed similar level of c-Jun as stage 1 tumor (Fig. 4L). It is known that stage 4s neuroblastoma patients have a high degree of spontaneous regression and favorable prognosis [55, 56]. Our analyses of four independent datasets suggest that c-Jun is a potential favorable prognostic factor for neuroblastoma.

### c-Jun can interact with CDC16 and inhibit anaphase-promoting complex formation

**Fig. 5:**
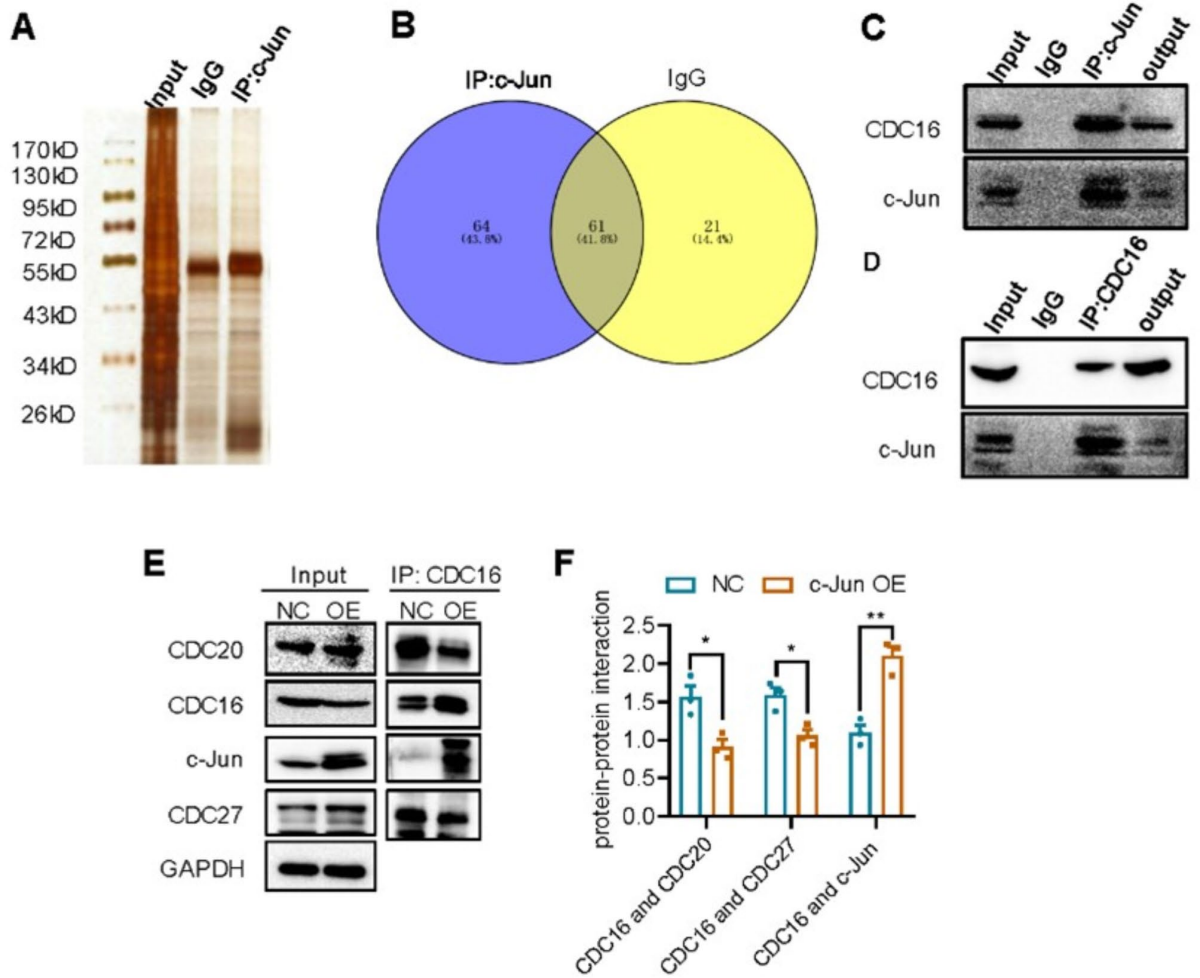
c-Jun interacted with CDC16 and inhibited its interaction with CDC20 and CDC27. **(A).** Proteins interacting with c-Jun were detected by co-immunoprecipitation (Co-IP) and silver staining in c-Jun OE cells. **(B).** Different protein bands in IgG group (NC) and c-Jun antibody group (IP) were analyzed by mass spectrometry, and the number of different proteins in the two groups were shown by Venn diagram. (**C-D**). Co-IP assay with c-Jun antibody (C) and CDC16 antibody (D) to confirm the interaction of CDC16 with c-Jun. IgG were used as negative control. (**E**). Co-IP assay with CDC16 antibody to detect the interaction between CDC16 and c-Jun or the interaction between CDC16 and CDC20/CDC27 in negative control (NC, cells transfected with Lentiviral vector) and c-Jun OE SH-SY5Y cells. (**F**). Relative protein level precipitated by CDC16 antibody. The ratio of IP group to total protein was statistically analyzed by t test (*p < 0.05, **p < 0.01, ***p < 0.001) and the results were presented as mean ± SEM (*n* = 3)

To understand how c-Jun regulates the transformation of cell cycle and differentiation to alleviate tumor malignancy, we analyzed the proteins that interact with c-Jun by co-immunoprecipitation experiments (Co-IP) and mass spectrometry. After c-Jun overexpression, APC6/CDC16, an important component of the anaphase-promoting complex (APC), interacted with c-Jun (Fig. 5A-B). Co-IP assay also confirmed the interaction between c-Jun and CDC16 (Fig. 5C-D). Since CDC16 is an important component of the APC complex, we speculated that c-Jun might affect the progression of cell cycle and differentiation by regulating APC formation. In the APC complex, CDC16 forms a complex with CDC27 protein, which is also required for anaphase progression in the mammalian cells [57–59]. Therefore, we further evaluated changes in the interaction of CDC16 with CDC20 and CDC27. Our results showed that the interaction of CDC16 with CDC20 and CDC27 was significantly repressed and the interaction of CDC16 with c-Jun was significantly elevated in c-Jun overexpressed SH-SY5Y cells (Fig. 5E-F). This implicates that c-Jun may compete with CDC20 and CDC27 for interaction with CDC16 and abolish APC complex formation.

### c-Jun promotes the initiation of cell differentiation via CDC16

**Fig. 6:**
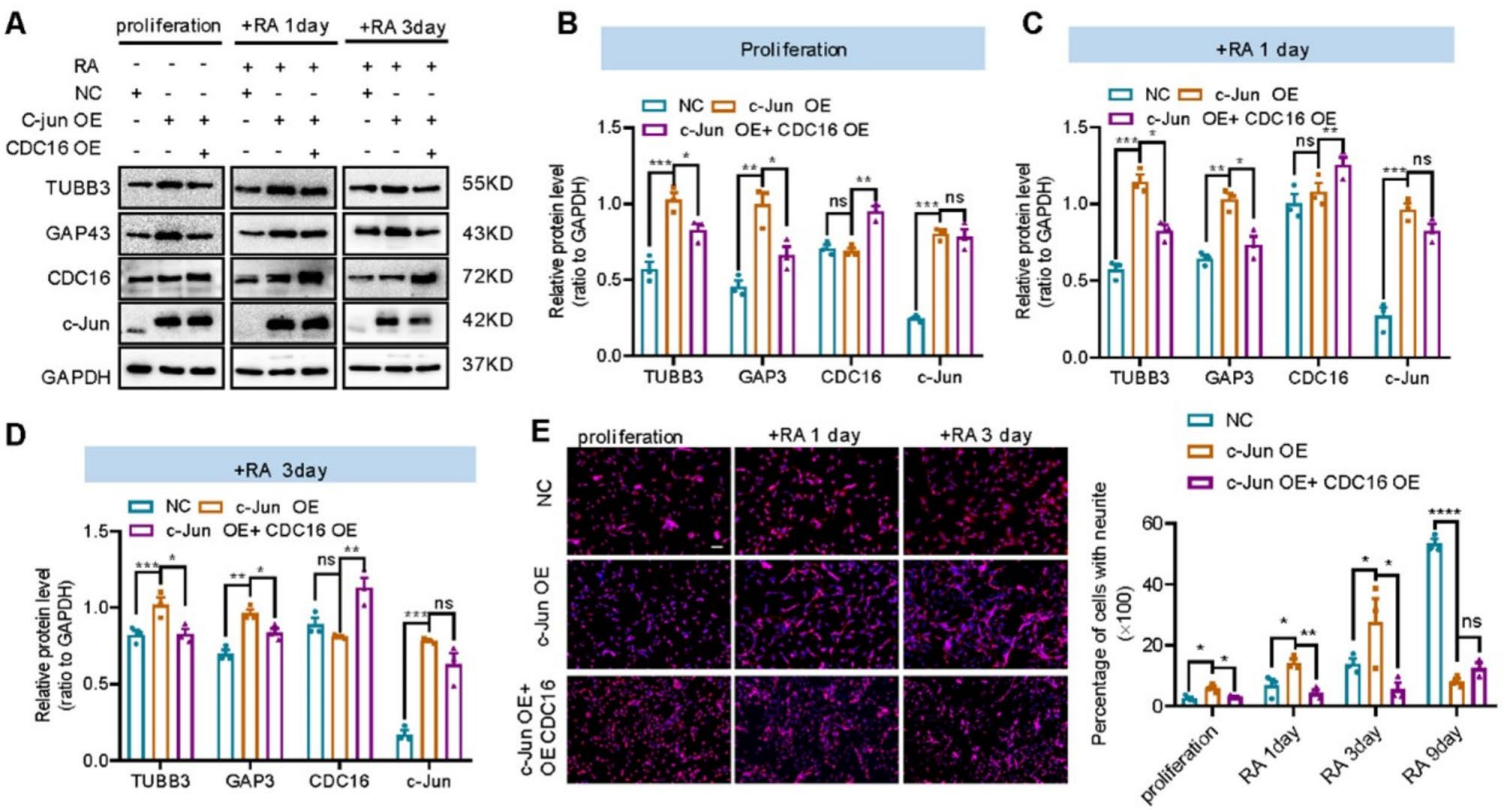
CDC16 overexpression abolished cell differentiation mediated by c-Jun overexpression. **(A)**. Immunoblots showing the expression of differentiation markers in control cells (NC), c-Jun overexpressed cells (c-Jun OE) and cells co-overexpressed c-Jun and CDC16 (c-Jun OE + CDC16 OE) after RA-induced differentiation. **(B-D).** Relative protein level of TUBB3 and GAP43 (ratio to GAPDH) analyzed by ordinary one-way ANOVA combined with Sidak’s multiple comparisons test. **(E).** Neural differentiation of NC, c-Jun OE and c-Jun OE + CDC16 OE SH-SY5Y cells. Cells were stained with TUBB3 and DAPI and images were acquired using a fluorescence microscope (Nikon). Number of cells with neurite outgrowth (> 20 μm) was counted using Image J software with cell counter plugin. Data were analyzed by ordinary one-way ANOVA combined with Sidak’s multiple comparisons test (mean ± SEM, *n* = 3; *p < 0.05, **p < 0.01, ***p < 0.001). Scale bar: 50 μm

To further explore whether CDC16 is involved in c-Jun mediated alteration of cell cycle and differentiation in SH-SY5Y cells, we overexpressed CDC16 in c-Jun overexpressed SH-SY5Y cells (c-Jun OE) and obtained a cell line with stable c-Jun and CDC16 expressions (c-Jun OE + CDC16 OE). Cell differentiation was induced by RA and BDNF (brain-derived neurotrophic factor) and examined by immunofluorescence with TUBB3 antibody. The expression of differentiation-related proteins was analyzed with Western blot and qPCR. Differentiation markers TUBB3 and GAP43 were upregulated in c-Jun OE cells but decreased to control level in c-Jun OE + CDC16 OE cells (Fig. 6A-D and Supplementary Fig. 3A-D). Furthermore, the number of cells with neurites was elevated in c-Jun OE cells and diminished in c-Jun OE + CDC16 OE cells (Fig. 6E). These results implicated that CDC16 was involved in c-Jun mediated cell differentiation.

**Fig. 7:**
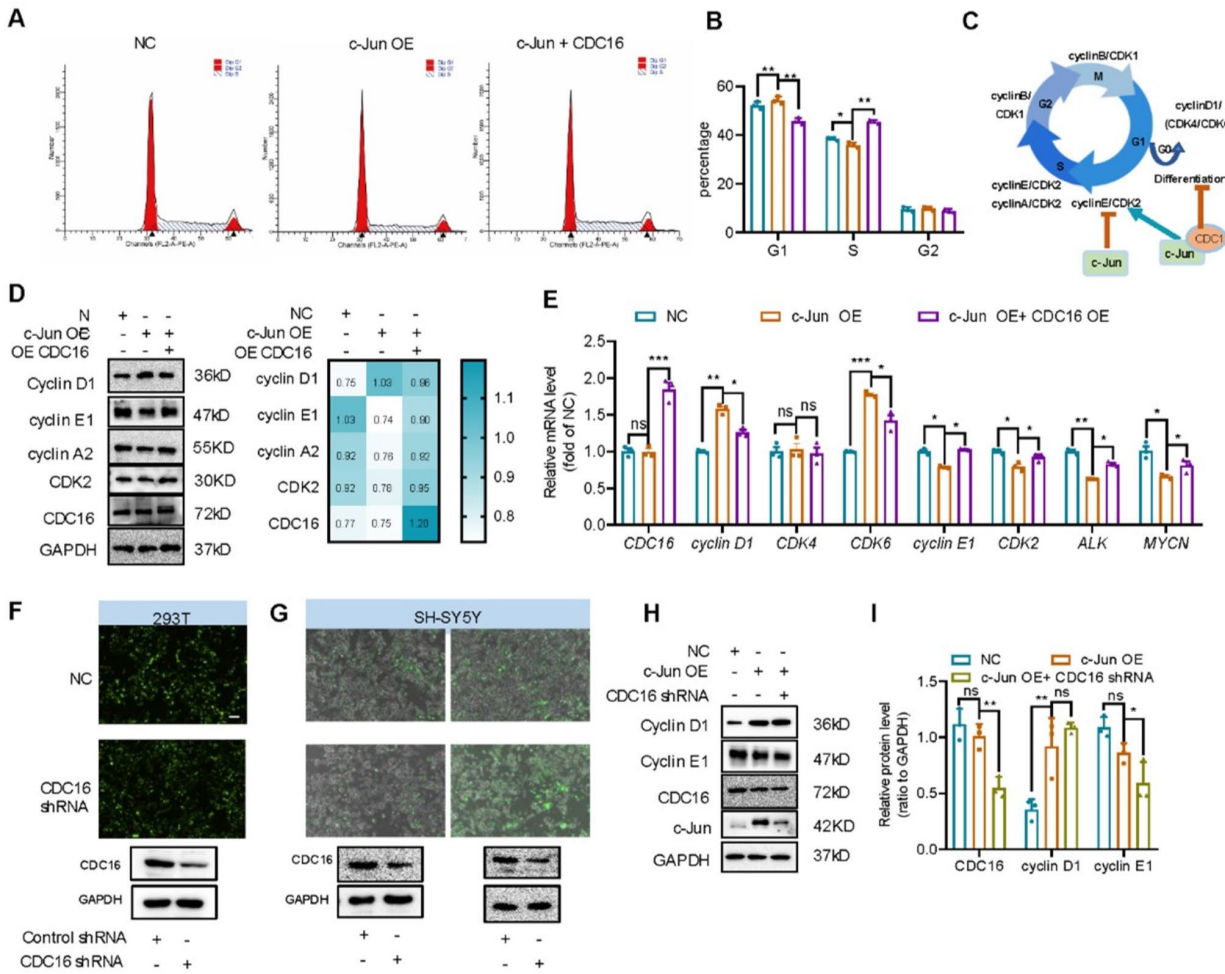
CDC16 overexpression rescued cell cycle arrest in G1 stage induced by c-Jun overexpression. **(A-B).** Cell cycle analysis by flow cytometry for negative control (NC), c-Jun OE and c-Jun OE + CDC16 OE cells. Percentage of cells at different cell cycle stage was analyzed with ordinary one-way ANOVA combined with Sidak’s multiple comparisons (mean ± SEM, *n* = 3; *p < 0.05, **p < 0.01). **(C).** Schematic representation of cell cycle progression and expression of cyclins at different stage. c-Jun overexpression inhibited cell cycle progression from G1 to S stage, increased G1 to G0 stage and promoted cell differentiation, whereas c-Jun and CDC16 co-overexpression promoted cell cycle progression. **(D).** Western blot showing the expression of cell cycle related markers in NC, c-Jun OE and C-Jun OE + CDC16 OE cells. Relative protein level was analyzed as ratio to GAPDH and heatmap was represented as mean ± SEM (*n* = 3). **(E).** qPCR result showing the mRNA expression of cell cycle related markers (*cyclin D1*,* cyclin E1*,* cyclin A2*,* cyclin B and CDKs*) in NC, c-Jun OE and c-Jun OE + CDC16 OE cells. Data were analyzed with ordinary one-way ANOVA combined with Sidak’s multiple comparisons (mean ± SD, *n* = 3; *p < 0.05, **p < 0.01, ***p < 0.001). **(F).** CDC16 shRNA was constructed to pLVX-shRNA2-GFP plasmid and then transfect into HEK293T cells. Cells expressing pLVX-shRNA2-GFP-CDC16 shRNA showed green fluorescence and expression of CDC16 was detected by Western blot. **(G).** Represent images of SH-SY5Y cells transfected with CDC16 shRNA lentivirus (left: 100µL and right: 200µL). **(H-I).** Western blot showing the expression of cell cycle related markers in NC, c-Jun OE and c-Jun OE + CDC16 knockdown cells. Relative protein level was analyzed as ratio to GAPDH (mean ± SEM, *n* = 3; *p < 0.05, **p < 0.01, ***p < 0.001)

To understand the role CDC16 plays in c-Jun mediated cell cycle arrest, we analyzed cell cycle changes between c-Jun OE and c-Jun OE + CDC16 OE cells using flow cytometry. Compared to c-Jun OE cells, overexpression of CDC16 reduced the percentage of cells arrested in the G1 phase from 70 to 50% (*p* < 0.01) and increased the percentage of cells arrested in S phase from 35 to 45% (*p* < 0.01) in c-Jun OE + CDC16 OE cells (Fig. 7A-B). This suggests that CDC16 overexpression abolished c-Jun overexpression-induced cell cycle arrest. Then, we analyzed the expression profile of cell cycle related genes. Compared to c-Jun OE cells, cyclin D1 expression was significantly repressed while cyclin E1 and cyclin A2 expression were re-increased in c-Jun OE + CDC16 OE cells (Fig. 7D-E). Thus, overexpression of CDC16 could rescue c-Jun overexpression mediated cell cycle arrest and cell cycle related protein expression. We further confirmed our observation by knocking down CDC16 in c-Jun OE cells with shRNA lentivirus and siRNA. shRNA lentivirus was constructed and produced in 293T cells, and the infection and knockdown efficiency of shRNA lentivirus was detected by Western blot (Fig. 7F-G). Knockdown of CDC16 did not modify c-Jun induced cyclin D1 upregulation and cyclin E1 downregulation in c-Jun OE cells (Fig. 7H-I). Thus, c-Jun can promote the differentiation of SH-SY5Y cells and inhibit cell cycle transition via negatively regulating CDC16.

### CDC16 overexpression abolishes the inhibitory effect of c-Jun on SH-SY5Y cell proliferation and migration

**Fig. 8:**
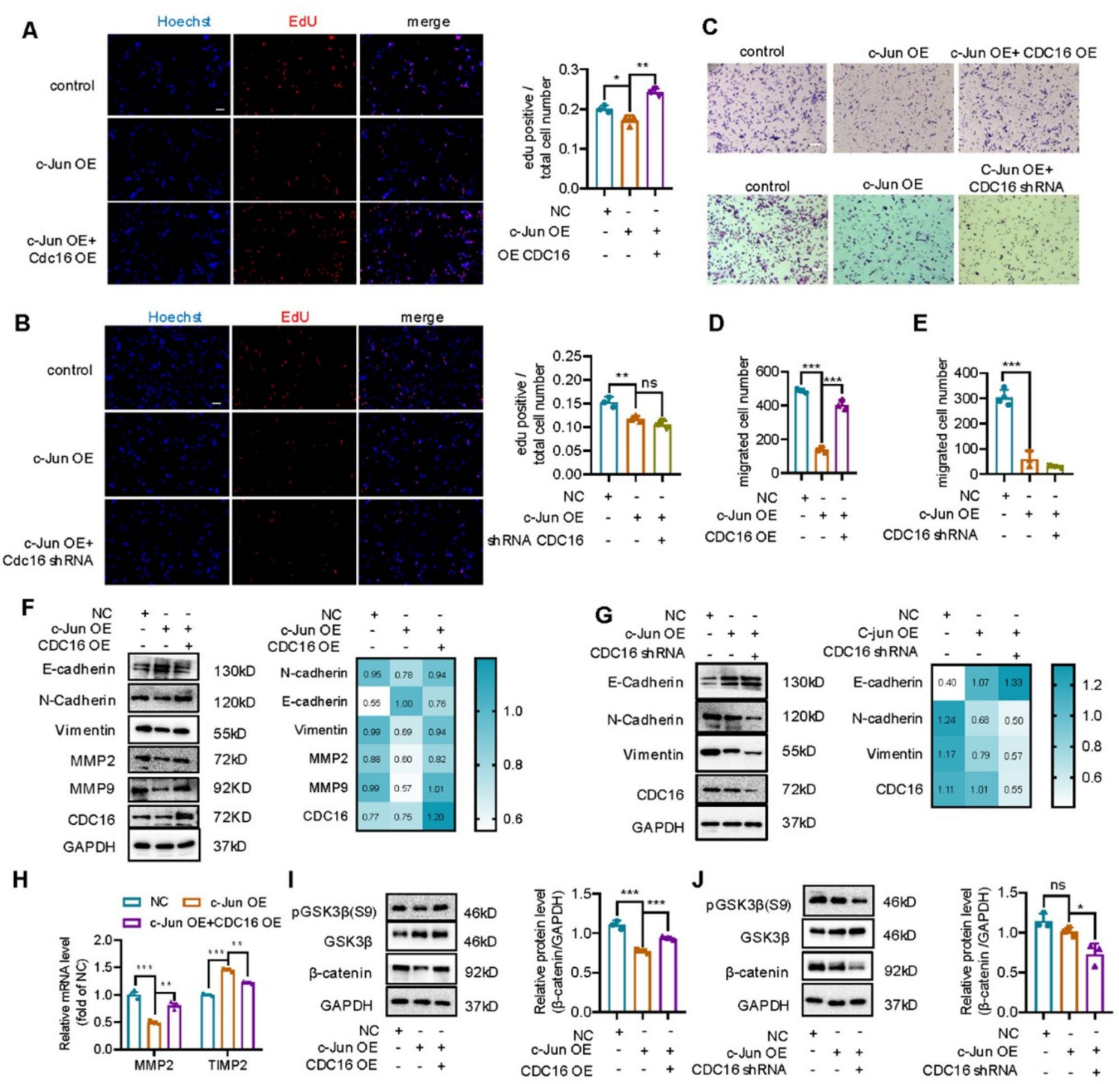
CDC16 overexpression abolished the inhibitory effect of c-Jun on SH-SY5Y cell proliferation and cell migration. **(A).** Proliferation of NC, c-Jun OE and c-Jun OE + CDC16 OE cells measured using EdU proliferation assay. **(B).** Proliferation of NC, c-Jun OE and c-Jun OE + CDC16 knockdown cells using EdU proliferation assay. Ratio of the number of EdU^+^ cells to the total number of cells was calculated from five areas in each well. The result was represented as mean ± SEM of three independent experiments. **(C-E).** Transwell assay to detect the effect of CDC16 on the inhibition of cell migration and invasion mediated by c-Jun overexpression. Numbers of migrated and invaded cells were calculated and analyzed statistically with ordinary one-way ANOVA combined with Sidak’s multiple comparisons test. **(F-G).** Protein immunoblotting assay to detect protein expression level of EMT and migration-related genes (N-cadherin, E-cadherin, vimentin, MMP9, MMP2 and TIMP2) in c-Jun OE + CDC16 OE cells (F) and c-Jun OE + CDC16 knockdown cells (G). Relative protein level was present as the ratio to GAPDH (mean ± SEM, *n* = 3). **(H).** qPCR to detect mRNA change of migration-related genes (*MMP2 and TIMP2*). **(I-J).** Immunoblot assay to detect phosphorylation level of GSK3β at ser9 and expression level of β-catenin in c-Jun OE cells (I, J), c-Jun + CDC16 OE cells (I) and c-Jun OE + CDC16 knockdown cells (J). Relative protein level was present as ratio to GAPDH. Data were statistically analyzed with ordinary one-way ANOVA combined with Sidak’s multiple comparisons test (*p < 0.05, **p < 0.01, ***p < 0.001). Scale bar = 100 μm

To identify how CDC16 affects the inhibitory effect of c-Jun on cell proliferation and migration, we either overexpressed or knocked down CDC16 in c-Jun OE SH-SY5Y cells and measured the respective impacts on proliferation using EdU proliferation assay and migration using Transwell assay. CDC16 overexpression re-increased the proportion of EdU + cells and migrated cells to the negative control level; whereas CDC16 knockdown by shRNA (CDC16 shRNA) further decreased the proportion of EdU + cells and migrated cells (Fig. 8A-E) was. This implicates that c-Jun overexpression may inhibit the proliferation and migration of SH-SY5Y cells via negatively regulating CDC16. We further examined the expression of EMT-related markers. CDC16 overexpression rescued the upregulation of E-cadherin and downregulation of N-cadherin and Vimentin mediated by c-Jun overexpression (Fig. 8F). The expression of MMP2 and MMP9, which play a key role in cell migration, was also rescued (Fig. 8F and H). CDC16 knockdown by shRNA and siRNA did not rescue and even enhanced the upregulation of E-cadherin and downregulation of N-cadherin and vimentin mediated by c-Jun (Fig. 8G and Supplementary Fig. 4A-C). Finally, we measured the phosphorylation level of GSK3β at ser 9 site (pGSK3β(s9)) and expression of β-catenin, which are key molecules in the wnt/β-catenin signaling pathway. CDC16 overexpression rescued the downregulation of β-catenin and pGSK3β(s9) (Fig. 8I-J) and CDC16 knockdown further reduced β-catenin and pGSK3β(s9) (Supplementary Fig. 4D-E). We conclude that c-Jun could inhibit the migration and invasion of SH-SY5Y cells by regulating EMT and/or wnt/β-catenin signaling pathway via inhibiting CDC16.

## Discussion

Our current study indicates that c-Jun is a key regulator of neuroblastoma differentiation. c-Jun expression is low in SH-SY5Y cells, but its expression can be increased in neuroblastoma cells upon RA-induced differentiation. We showed that c-Jun overexpression modified the expression of cell cycle related proteins, which are required for cell cycle arrest and neuronal differentiation in neuroblastoma cells. Its overexpression can also repress proliferation-promoting genes and activate neuronal differentiation genes under RA induced neuroblastoma cell differentiation. Thus, c-Jun may have tumor suppression effect in neuroblastoma. We found out high c-Jun expression is prognostic for favorable clinical outcome in four independent gene expression datasets of neuroblastoma tumors. c-Jun expression was correlated with histological type, risk grade, death from disease and clinical stage. Kaplan-Meier survival analyses showed that OS and EFS were better in neuroblastoma with high c-Jun expression. Surprisingly, stage 4s neuroblastoma expressed relatively high level of c-Jun. Since stage 4s patients have a high degree of spontaneous regression [55, 56], c-Jun is likely to play a role in the spontaneous differentiation and regression of neuroblastoma. This finding suggests that c-Jun not only promotes differentiation and suppresses tumor malignancy but also may enhance the efficacy of existing treatment modalities, such as anti-GD2 immunotherapy which is a cornerstone of high-risk neuroblastoma treatment [3, 60]. Heitzeneder et al. showed that c-Jun OE improves GPC2-CAR T cell potency and mediates durable antitumor activity against NBs [22]. Lynn et al. provided evidence that c-Jun overexpression in GPC2-CAR T cells results in enhanced tumor control in preclinical models of neuroblastoma, without inducing significant toxicity [23]. By modulating the differentiation status and immune microenvironment of neuroblastoma cells, c-Jun could serve as a valuable therapeutic target to complement current immunotherapy strategies.

Although c-Jun was previously shown to have a role in proliferation and differentiation of neuroblastoma cells [26, 27, 61], its functional mechanism has never been fully elucidated. Cells entering resting state and differentiation interfere with the process of cell cycle, and the cell cycle will be inhibited by the upcoming differentiation, hindering cell proliferation [39, 62]. In this study, we showed that c-Jun inhibited cell cycle progression in SH-SY5Y cells and identified CDC16 was co-immunoprecipitated by c-Jun and downregulate in c-Jun OE SH-SY5Y cells. CDC16 is a major component of the TPR subcomplex of anaphase-promoting complex (APC/C) and plays an important role in cell cycle progression and cell proliferation [63–65]. CDC20, a co-activator of APC/C, is already present during interphase [66]. Our results demonstrated that c-Jun overexpression repressed the interaction between CDC16 and CDC20. Thus, we speculate that c-Jun inhibits cell cycle progression and promotes initiation of cell differentiation by competitively binding to CDC16, which, in turn, inhibits the formation of APC/C-CDC20 complex. Since interaction of CDC16 or CDC27 with certain other proteins inhibits substrate binding to APC/C which regulates the timing of mitosis [67], the interaction between c-Jun and CDC16 may also inhibit substrate binding to APC/C and repress cell cycle progression. We confirmed this by observation that CDC16 knockdown increased the number of cells in G1/G0 phase whereas CDC16 overexpression decreased the number of cells in G1/G0 phase and promoted G1/S transition.

APC/C was originally discovered as an E3 ubiquitin ligase responsible for degradation of mitotic cyclins [68]. APC/C activity is crucial for the exit from mitosis. Cyclins are produced and degraded in a cell cycle-dependent manner and bind to specific CDKs to form active complexes during specific stages of the cell cycle [69, 70]. In c-Jun OE SH-SY5Y cells, we observed that expressions of cyclin D1 and CDK6 were increased but expressions of cyclin E1 and CDK2 were decreased. CDC16 overexpression rescued the effect of c-Jun on cyclins. The cyclin D1-CDK4/6 complex is produced during the G1 phase and responsible for driving the cell cycle past the G1 checkpoint [71] and the cyclin E-CDK2 complex is produced during the G1/S transition and required for the initiation of DNA replication [72]. Furthermore, cyclin A2 and CDK2, produced during S phase and required for progression of DNA replication and transition into mitosis [73], were downregulated in c-Jun OE SH-SY5Y cells. By inhibiting APC/C complex formation, CDC16 manipulation likely stabilizes cyclin D and other APC/C substrates, leading to cell cycle arrest at the G1 phase. The stabilization of cyclin D, due to impaired APC/C activity, may disrupt normal cell cycle progression and promote cell differentiation. Cyclin D accumulation has been shown to induce cell cycle exit and initiate differentiation programs in various types of cells including neuroblastoma [74]. We also observed that c-Jun overexpression inhibited the expression of p21, a cyclin-dependent kinase inhibitor controlling G1/S transition and G1 to S phase arrest [75]. All these observations suggest that c-Jun inhibits G1/S transition and induces cell cycle arrest at G1 phase whereas CDC16 promotes cell cycle progression in neuroblastoma cells.

Transcription factors play a vital role in the regulation of gene expression. As a transcription factor, c-Jun regulated the expressions of N-cadherin, E-cadherin, MMP2, MMP9 and TIMP2, which are involved in EMT and regulate migration and invasion of most cancers [51]. EMT is a critical process in neuroblastoma that contributes to tumor plasticity, invasion, and metastasis. Neuroblastoma exhibits a biphasic nature with tumors composed of adrenergic (ADRN) and mesenchymal (MES) subtypes. The ADRN subtype is characterized by high expression of adrenergic markers (e.g., PHOX2B, TH), whereas the MES subtype exhibits features of mesenchymal differentiation (e.g., Vimentin, fibronectin-1) [76]. RA treatment induces a transition from the ADRN to the MES phenotype, which is associated with increased tumor plasticity and therapy resistance [76, 77]. Key EMT markers, such as Vimentin, SNAI1, and ZEB1, are upregulated in the MES subtype and associated with poor prognosis [76, 78]. c-Jun, as a component of the AP-1 transcription factor complex, has been implicated in regulating EMT-related genes (such as N-cadherin, E-cadherin, Vimentin) [79]. Herein, we showed that c-Jun overexpression inhibited Vimentin expression, further supporting its role in neuroblastoma differentiation and transition from the ADRN to the MES phenotype. c-Jun also inhibits the expression of β-catenin in nucleus. β-catenin is downstream of wnt/β-catenin signaling pathway that regulates cancer cell proliferation and migration [20]. Wnt/β-catenin axis has emerged as a versatile modulator of EMT [80]. Aberrant activation of Wnt ligands/receptors in Wnt signaling pathway promotes progression and relapse of NB by regulating cell proliferation and metastasis [54]. Despite downregulation of GSK3β phosphorylation at ser9 site, the molecular mechanism of c-Jun-induced β-catenin downregulation remains elusive and further studies are warranted.

In summary, c-Jun contributes to RA induced neuroblastoma differentiation through two distinct mechanisms: (1) its transcriptional activity, which regulates the expression of differentiation-related genes and cell cycle inhibitors, and (2) its interaction with CDC16, which is key component in APC complex, regulate cell cycle regulators and enhances G1 arrest. Via interacting with CDC16, it can inhibit proliferation and promote differentiation of neuroblastoma cells. These mechanisms suggest that c-Jun could enhance the sensitivity of neuroblastoma cells to RA and improve the efficacy of RA-based therapies. RA resistance is a significant challenge in neuroblastoma therapy [5, 9, 10], and our findings suggest that c-Jun may play a role in addressing this issue by amplifying the transcriptional activation of differentiation-related genes and enhance cell differentiation. Our current study provides a new perspective for neuroblastoma research and demonstrates a theoretical basis for developing novel anti-tumor therapeutics targeting c-Jun. Future studies are warranted to explore the potential of combining RA with c-Jun activation strategies to overcome RA resistance and achieve more durable therapeutic outcomes.

### Limitations of our current study

Our current study has several limitations that should be acknowledged. First, we only utilized the SH-SY5Y cell line, which may limit the generalizability of our findings. Different neuroblastoma cell lines may exhibit heterogeneous responses to c-Jun modulation, and further validation in additional cell lines is necessary. Secondly, we demonstrated at the cellular level that c-Jun promotes differentiation and inhibits cell cycle progression, thereby suppressing the malignant progression of neuroblastoma, however, further in vivo studies are required to confirm that c-Jun can indeed inhibit neuroblastoma growth in a physiological setting. Mouse tumor in situ model could be used to confirm the therapeutic potential of c-Jun targeting. Thirdly, although we found that c-Jun influences the differentiation process by regulating the transcription and expression of differentiation- and cell cycle-related genes and further inhibits cell cycle progression by interacting with CDC16 to suppress APC complex formation, the specific interaction sites and detailed mechanisms between c-Jun and CDC16 require further investigation. Lastly, our current study relies on permanent rather than inducible overexpression of c-Jun and CDC16, which may not fully recapitulate the dynamic regulation of these proteins in physiological or pathological contexts. Despite these limitations, our findings provide valuable insights into the role of c-Jun in neuroblastoma differentiation and cell cycle regulation, laying the groundwork for future studies.

## Electronic supplementary material

Below is the link to the electronic supplementary material.

Supplementary Tables

Supplementary Fig.1

Supplementary Fig.2

Supplementary Fig.3

Supplementary Fig.4

Supplementary Figure Legend

## Data Availability

No datasets were generated or analysed during the current study.
