# Supplementary material for: c-Jun promotes neuroblastoma cell differentiation by inhibiting APC formation via CDC16 and reduces neuroblastoma malignancy
Source: Biol Direct. 2025 Mar 27;20:37. doi: 10.1186/s13062-025-00630-1 (PMC11948754; doi:10.1186/s13062-025-00630-1)
Supplement: Supplementary file 1 — Supplementary Tables [file 13062_2025_630_MOESM1_ESM.docx]

| **Name** | **sequence** |
| --- | --- |
| c-Jun shRNA | GGATCCG GCAAACCTCAGCAACTTCA TCAAGAG  TGAAGTTGCTGAGGTTTGC TTTTTTGAATTC |
| CDC16 shRNA | GGATCCG GGACGAAAGTGGCTTCAAA TCAAGAG  TTTGAAGCCACTTTCGTCC TTTTTTGAATTC |
| CDC16 SiRNA-1 | GGACGAAAGUGGCUUCAAA |
| CDC16 SiRNA-2 | GCAGACAUUAAAGACAAAU |

Supplementary Table 1. shRNA and siRNA sequence

Supplementary Table 2. Primer pairs in this study

| **Primer** | **DNA Sequence (5'-3')** |
| --- | --- |
| CDC16 For | ACACAAGGAACTCTTCGACG |
| CDC16 Rev | GCCAGGTTCAAAGCGAAATG |
| CyclinD1For | TGGGCAGGTATTACGAGACTG |
| CyclinD1 Rev | ACTCCCGCTTATACTGGGCTA |
| CyclinE1 For | TGGACAGTTACGCGCACAT |
| CyclinE1 Rev | CGAGTAGGACATGCTGTAGGT |
| CyclinB For | CGCCTACGAGACGCTGTATG |
| CyclinB Rev | TGCTTGAGTGAGAAGTTGCCA |
| CyclinA2 For | CAAGCACGAAGCCAACGAC |
| CyclinA2 Rev | CTCCATCCCGCATCTCCAC |
| CDK1 For | GTAAATTTGCTTCTGGCCTTCC |
| CDK1 Rev | ATTCCCTCACTCTCATCAGG |
| CDK2 For | GCTGCAAAACTTCTTCCCTC |
| CDK2 Rev | AGTAGGTCTCATCCGTGTT |
| Continue Supplemental Table 2. Primer pairs in this study | |
| **Primer** | **DNA Sequence (5'-3')** |
| CDK4 For | TTTTAGGGAGCGAGCAGGAA |
| CDK4Rev | GCGTTGCCTTAGCACTTCTTT |
| CDK6 For | CAAGGAGATTGGGGACAA |
| CDK6 Rev | TTGCTTTGAGTCACACTGGT |
| c-jun For | TACAGTCTGGACTTGTGTTGC |
| c-jun Rev | TAGCCCCCAACCTCTTTGCT |
| PCNA For | CTGAGGGCTTCGACACCTAC |
| PCNA Rev | TCACTCCGTCTTTTGCACAG |
| MMP-2 For | TGATCTTGATCTTCATTGTG |
| MMP-2 Rev | AAAACCGCAGTGGGGTCACATC |
| MMP-9 For | GGACGGGCTCCTGGCACAC |
| MMP-9 Rev | GAGCGGCCCTCGAAGATGAAG |
| MYCN For | TGATCCTCAAACGATGCCTTC |
| MYCN Rev | GGACGCCTCGCTCTTTATCT |
| TIMP2 For | AAGCGGTCAGTGAGAAGGAAG |
| TIMP2 Rev | GGGGCCGTGTAGATAAACTCTAT |
| ALK For | TGGTTGCTTTTGCTGGGGTA |
| ALK Rev | GAGACATCTACAGGGCGAGC |
| ACTB For | TTGCCCTGAGGCTCTTTTCC |
| ACTB Rev | TCCTTCTGCATCCTGTCAGC |
