# Supplementary figures and images for: c-Jun promotes neuroblastoma cell differentiation by inhibiting APC formation via CDC16 and reduces neuroblastoma malignancy
Source: Biol Direct. 2025 Mar 27;20:37. doi: 10.1186/s13062-025-00630-1 (PMC11948754; doi:10.1186/s13062-025-00630-1)

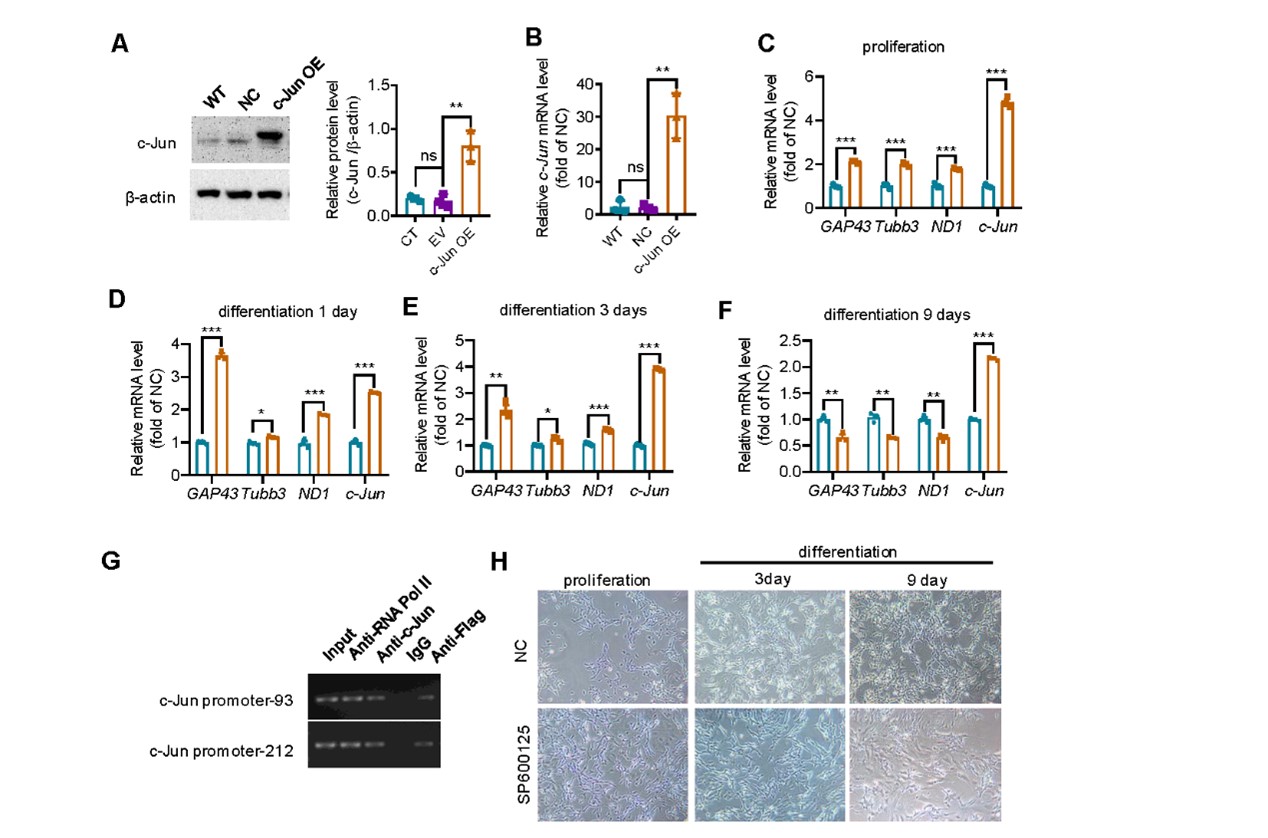

Supplement: Supplementary file 2 — Supplementary Fig.1 [file 13062_2025_630_MOESM2_ESM.jpg]

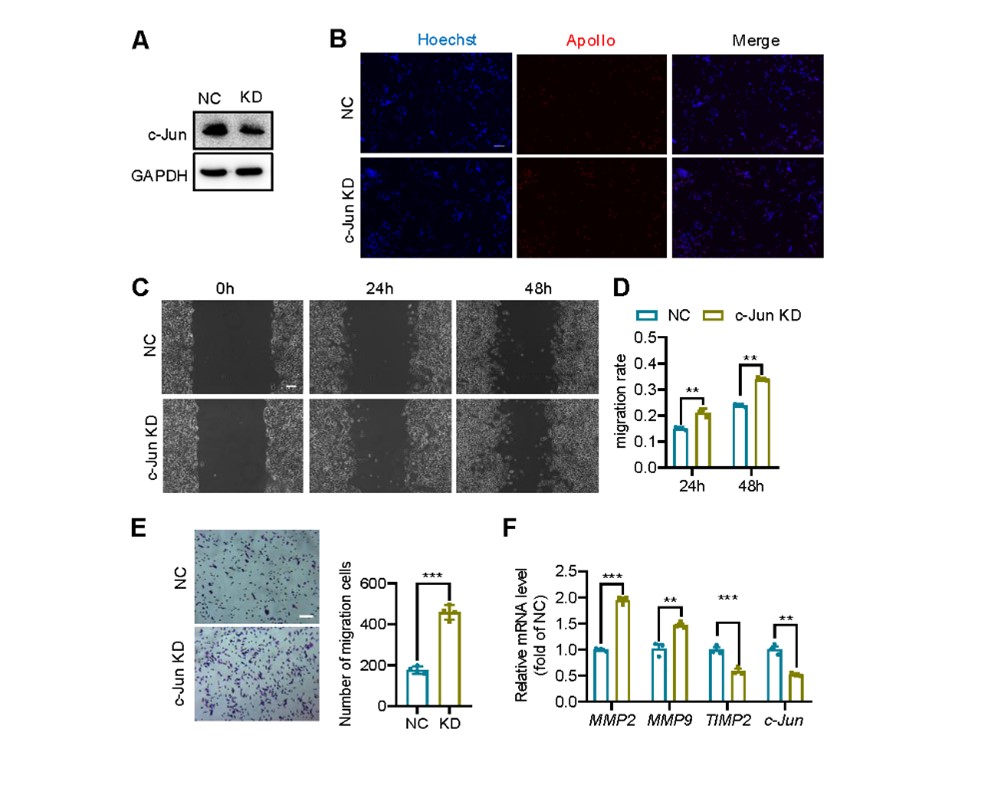

Supplement: Supplementary file 3 — Supplementary Fig.2 [file 13062_2025_630_MOESM3_ESM.jpg]

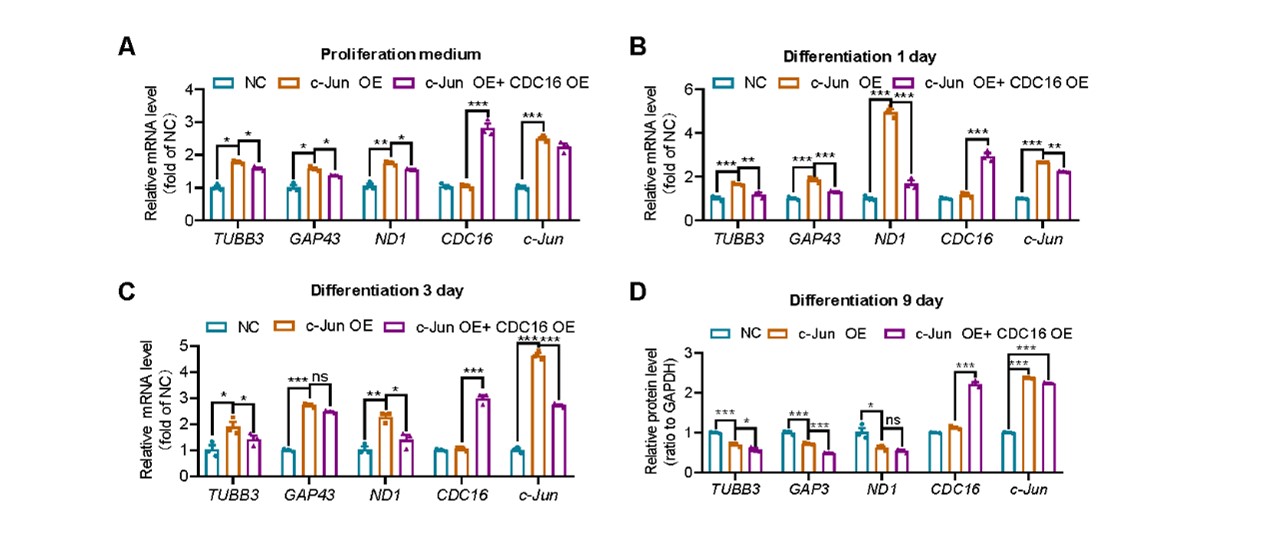

Supplement: Supplementary file 4 — Supplementary Fig.3 [file 13062_2025_630_MOESM4_ESM.jpg]

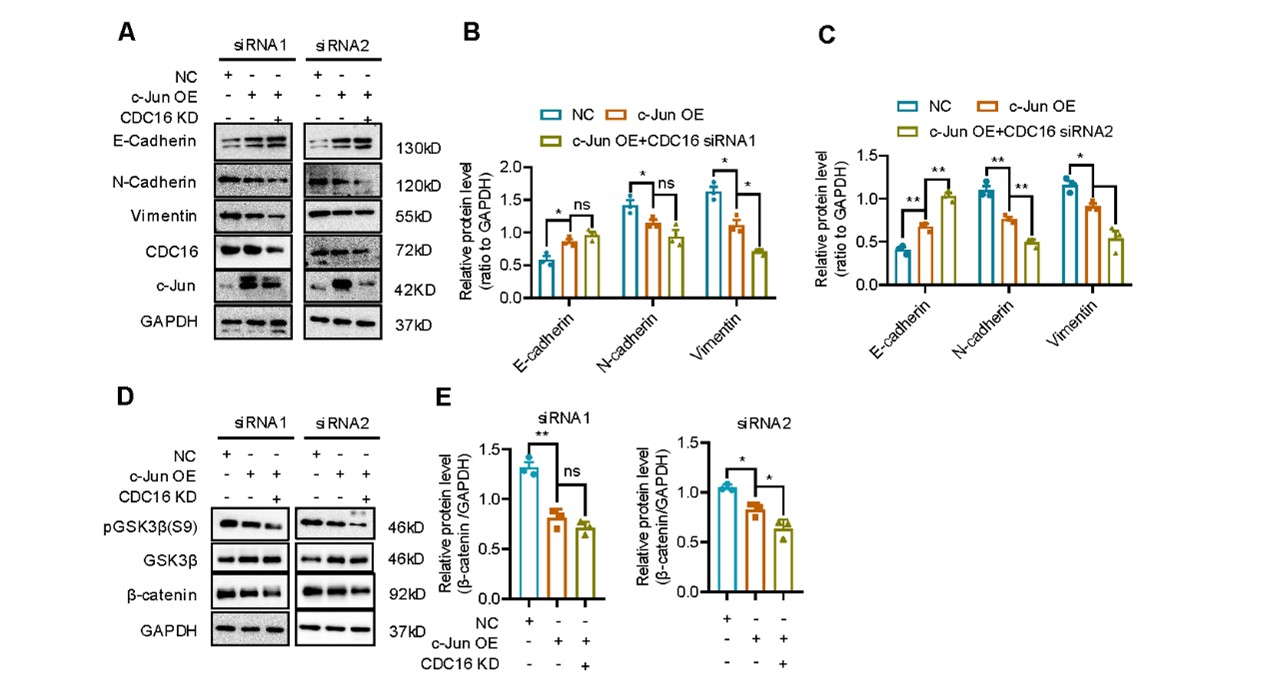

Supplement: Supplementary file 5 — Supplementary Fig.4 [file 13062_2025_630_MOESM5_ESM.jpg]
