# Supplementary material for: c-Jun promotes neuroblastoma cell differentiation by inhibiting APC formation via CDC16 and reduces neuroblastoma malignancy
Source: Biol Direct. 2025 Mar 27;20:37. doi: 10.1186/s13062-025-00630-1 (PMC11948754; doi:10.1186/s13062-025-00630-1)
Supplement: Supplementary file 6 — Supplementary Figure Legend [file 13062_2025_630_MOESM6_ESM.docx]

**Legends of supplementary figures**

**Supplementary Figure 1.** c-Jun overexpression promoted differentiation of SH-SY5Y cells. (**A-B).** Construction of c-Jun overexpressed cell line (c-Jun OE). (A). Immunoblot assay to detect protein expression of c-Jun in negative control (NC) and c-Jun OE cells; and (B). qPCR to detect mRNA level of c-Jun in NC and c-Jun OE cells. Relative protein and mRNA levels of c-Jun (ratio to β-actin) were analyzed by unpaired t-test (mean ± SEM, n =3; **p* < 0.05, ***p* < 0.01). **(C-F).** qPCR assay showing the expression of differentiation markers (*GAP43*, *Tubb3* and *ND1*) in NC) and c-Jun OE cells after RA-induced differentiation. **(G).** Ch-IP assay by using c-Jun antibody to detect the binding of c-Jun onto the promoter of c-Jun. IgG and anti-RNA polymerase II antibody were used as negative and positive controls, respectively. **(H).** Neural differentiation of NC and c-Jun inhibitor treated SH-SY5Y cells.

**Supplementary Figure 2.** Knockdown of c-Jun expression by shRNA (c-Jun KD) promoted proliferation and migration of SH-SY5Y cells. **(A).** Immunoblot to detect the expression of c-Jun after cells infected with c-Jun shRNA (c-Jun KD). **(B).** Proliferation of NC and c-Jun OE cells as measured with the EdU proliferation assay. **(C).** Effect of c-Jun KD on the wound healing ability of SH-SY5Y cells as detected by cell scratch experiment. The migration rate (ratio of migrated area to originally scratched area) was statistically analyzed by unpaired *t*-test. **(E).** Transwell assay to detect the effect of c-Jun KD on cell migration. Number of migrated cells was calculated and statistically analyzed by unpaired *t*-test. **(F).** qPCR to detect mRNA changes of migration-related genes (*MMP9, MMP2 and TIMP2*) in NC and c-Jun KD cells. The qPCR results were calculated using 2^-ΔΔCT^ and analyzed with unpaired *t*-test using GraphPad software (**p* < 0.05, ***p* < 0.01, ****p* < 0.001). The data were presented as mean ± SEM (n=3).

**Supplementary Figure 3.** qPCR assay to detect mRNA changes of differentiation related genes (*TuBB3, GAP43 and ND1*) in NC, c-Jun OE and c-Jun OE+CDC16 OE SH-SY5Ycells at different condition: **(A).** cells cultured in growth medium, **(B).** cells induced to differentiate for 1 d with RA, **(C).** cells induced to differentiate for 3 d with RA, and **(D).** cells induced to differentiate for 9 d with RA and BDNF. qPCR results were calculated using 2^-ΔΔCT^ and analyzed with one-way ANOVA combined with Sidak’s multiple comparisons using GraphPad software (**p* < 0.05, ***p* < 0.01, ****p* < 0.001). The data presented as mean ± SEM (n=3).

**Supplementary Figure 4.** **(A).** Immunoblot assay to detect protein expression level of EMT and migration-related proteins (N-cadherin, E-cadherin, vimentin, MMP9, MMP2 and TIMP2) in c-c-Jun OE+CDC16 KD. **(B-C).** Relative protein level was presented as the ratio to GAPDH (mean ± SEM, n=3). **(D).** Immunoblot assay to detect the phosphorylation level of GSK3β at ser9 and expression level of β-catenin in cells c-Jun OE and c-Jun OE+CDC16 KD cells. **(E).** Relative protein level was presented as the ratio to GAPDH. All data were statistically analyzed with ordinary one-way ANOVA combined with Sidak’s multiple comparisons test, **p* < 0.05, ***p* < 0.01, ****p* < 0.001. NC: SH-SY5Y cells infected with control virus; c-Jun OE: SH-SY5Y cells infected with c-Jun overexpression lentivirus; and c-Jun OE +CDC16 KD: SH-SY5Y cells infected with c-Jun overexpression lentivirus and transfected with CDC16 siRNA.
